# The Recovery Rate in Mental Hospitals

**Published:** 1912-11-02

**Authors:** A. R. Urquhart

**Affiliations:** James Murray Royal Asylum, Perth.


### The Editor's Letter-Box.

### The Recovery Rate in Mental Hospitals.

*To the Editor of* The Hospital.

Sir,—I have just read your article on "The Recovery Rate in Mental Hospitals" in your issue for October 26. It reminds me of my proposal as to how the recovery rate might be returned in the statistical tables of the Medico-Psychological Association, agreed upon with many dissentients in 1905-6. I then urged that the terms should be defined as a preliminary to their being put into use; but unfortunately failed to induce the Association to come to any agreement on these fundamental and important questions, which, in my opinion, still leave the statistics of asylums notoriously untrustworthy and impossible in collation. There is, for instance, apparently no possibility of general agreement as to the use of the word "Recoveries," by which I mean those in whom there is re-establishment of mental soundness permitting of return to ordinary life without need of the care and supervision of others. A final, permanent recovery is just as rare in insanity as a true recovery from gout. But there would appear to be a determination to use the word "recovery" in a much looser sense than I have indicated. For instance, I remember one distinguished physician urging that it must be elastic enough to include the state of an asylum patient who had been discharged as recovered to her husband's care because the physician was assured that the husband would so manage her that she might with his aid remain at home. That would certainly not represent a recovery to me. It is evident that while the word "recovery" is used in such a wide sense there must be the absurd discrepancies which have continued since 1869, and will continue indefinitely until there is agreement in definitions, fixed and determined by authority. In the present usage it is not so long ago since an American asylum published such a startling return of success in treatment that it really reduced the statistics to absolute absurdity. I would suggest, however, that inquiry should be made regarding recoveries from the medical side of general hospitals as well as from asylums. I think that it would be found that the recovery rate is much the same in both classes of institution—namely, about 30 per cent.

A great deal of the returns made by physicians in asylums is set forth with very imperfect knowledge of the factors. For instance, you refer to alcoholic statistics. What is fixed and determined in my experience of patients who have come here as habitual drunkards is that they are originally and fundamentally insane persons, and not drunkards first and secondly insane. While the facts submitted are so elusive and so misrepresented, and while they are gathered into collections which cannot be definitely described, we are not in a position to defend such figures as you set forth in last week's Hospital. I have already pointed out in the Morison Lectures of 1907 that when asylum recoveries are referable to persons, not to cases, they are declared only so far as official statistics can show. No doubt the same remark may be made regarding the medical results of general hospitals dealing with other constitutional diseases of obscure causation. The persons are received and treated and discharged cured, and later return relapsed. The vital history can be completed only after death.

I suppose that it is impossible to expect a truer recovery rate until some successful attempt is made to improve the conditions under which we have to work.—Yours faithfully,

A. R. Urquhart.

James Murray Royal Asylum, Perth.

